# The Predictive Utility of MammaPrint and BluePrint in Identifying Patients with Locally Advanced Breast Cancer Who are Most Likely to Have Nodal Downstaging and a Pathologic Complete Response After Neoadjuvant Chemotherapy

**DOI:** 10.1245/s10434-023-14027-9

**Published:** 2023-09-01

**Authors:** Peter Blumencranz, Mehran Habibi, Steve Shivers, Geza Acs, Lisa E. Blumencranz, Erin B. Yoder, Bastiaan van der Baan, Andrea R. Menicucci, Patricia Dauer, William Audeh, Charles E. Cox

**Affiliations:** 1 BayCare Oncology, Clearwater, FL USA; 2 Johns Hopkins University, Baltimore, MD USA; 3 Comprehensive Breast Cancer Program, H. Lee Moffitt Cancer Center & Research Institute, Tampa, FL USA; 4 Department of Pathology, H. Lee Moffitt Cancer Center and Research Institute, Tampa, FL USA; 5 Agendia, Inc., Irvine, CA USA; 6 Agendia, NV, Amsterdam, The Netherlands

**Keywords:** Nodal downstaging, Axillary staging, 70-gene signature, 80-gene signature, Surgical management

## Abstract

**Background:**

Neoadjuvant chemotherapy (NCT) increases the feasibility of surgical resection by downstaging large primary breast tumors and nodal involvement, which may result in surgical de-escalation and improved outcomes. This subanalysis from the Multi-Institutional Neo-adjuvant Therapy MammaPrint Project I (MINT) trial evaluated the association between MammaPrint and BluePrint with nodal downstaging.

**Patients and Methods:**

The prospective MINT trial (NCT01501487) enrolled 387 patients between 2011 and 2016 aged ≥ 18 years with invasive breast cancer (T2–T4). This subanalysis includes 146 patients with stage II–III, lymph node positive, who received NCT. MammaPrint stratifies tumors as having a Low Risk or High Risk of distant metastasis. Together with MammaPrint, BluePrint genomically (g) categorizes tumors as gLuminal A, gLuminal B, gHER2, or gBasal.

**Results:**

Overall, 45.2% (*n* = 66/146) of patients had complete nodal downstaging, of whom 60.6% (*n* = 40/66) achieved a pathologic complete response. MammaPrint and combined MammaPrint and BluePrint were significantly associated with nodal downstaging (*p* = 0.007 and *p* < 0.001, respectively). A greater proportion of patients with MammaPrint High Risk tumors had nodal downstaging compared with Low Risk (*p* = 0.007). When classified with MammaPrint and BluePrint, more patients with gLuminal B, gHER2, and gBasal tumors had nodal downstaging compared with HR+HER2−, gLuminal A tumors (*p* = 0.538, *p* < 0.001, and *p* = 0.013, respectively).

**Conclusions:**

Patients with genomically High Risk tumors, defined by MammaPrint with or without BluePrint, respond better to NCT and have a higher likelihood of nodal downstaging compared with patients with gLuminal A tumors. These genomic signatures can be used to select node-positive patients who are more likely to have nodal downstaging and avoid invasive surgical procedures.

**Supplementary Information:**

The online version contains supplementary material available at 10.1245/s10434-023-14027-9.

Neoadjuvant chemotherapy (NCT) has been increasingly used in early stage and locally advanced breast cancers to monitor a patient’s response to treatment.^1^ NCT can also reduce a patient’s primary tumor burden and extent of nodal involvement, which may increase the likelihood of breast-conserving surgery and targeted axillary dissection (TAD), reducing surgical morbidity and improving long-term outcomes.^2,3^ Patients with non-Luminal A subtypes who achieve a pathologic complete response (pCR) of the primary tumor and nodes are associated with superior outcomes compared with those with any residual disease.^3–7^ A complete response in the breast correlates with a complete axillary response, and patients who have a breast-only or axilla-only complete response have better long-term outcomes compared with those with no response to NCT.^3,4^

Given the differential outcomes in patients achieving nodal downstaging, it is important to assess nodal responses for surgical planning.^7–9^ Axillary lymph node dissection (ALND) provides accurate nodal staging information but has increased associated morbidities, most notably, lymphedema.^10,11^ Sentinel lymph node biopsies (SLNB), which are less invasive and can mitigate the need for ALND and its associated morbidities, have become the standard of care for patients presenting with node-negative disease or patients who present with clinically node-positive disease and are downstaged following NCT.^10–12^ However, SLNB can have high false negative rates, leaving the management of clinically node-positive patients debatable.^10,13^ More recently, targeted axillary dissection (TAD), which incorporates SLNB and radio- or carbon-tracing to selectively remove positive nodes, has been increasingly utilized by some surgeons to improve the accuracy of axillary staging without compromising clinical outcome in clinically node-positive patients.^14,15^

Here, we considered whether genomic information from the breast primary tumors could be used to identify patients who are likely to have nodal downstaging with NCT and thus be good candidates for less invasive nodal staging procedures, such as TAD. MammaPrint, a 70-gene signature (70-GS) that classifies patients as having a High Risk or Low Risk of distant metastasis, provides predictive and prognostic information for patients with early stage breast cancer and can identify Low Risk patients who may safely avoid chemotherapy.^16,17^ BluePrint is an 80-gene molecular subtyping signature that genomically (g) categorizes tumors as Luminal-Type (gLuminal), human epidermal growth factor 2 (HER2)-Type (gHER2), or Basal-Type (gBasal) on the basis of underlying signaling pathways.^18^ Together, MammaPrint and BluePrint identify molecular subtypes that more precisely determine therapeutic benefit in patients with early stage breast cancer than clinical factors alone, and this genomic classification corresponds better with 5-year outcomes and neoadjuvant chemosensitivity.^19,20^

The Multi-Institutional Neo-adjuvant Therapy MammaPrint Project I (MINT) trial was designed to evaluate the ability of MammaPrint and BluePrint to predict responsiveness to NCT in patients with locally advanced breast cancer. In this post hoc subanalysis from the MINT trial, we assessed the ability of MammaPrint and BluePrint to determine the likelihood of nodal downstaging after NCT.

## Patients and Methods

### Patient Cohort

**Fig. 1:**
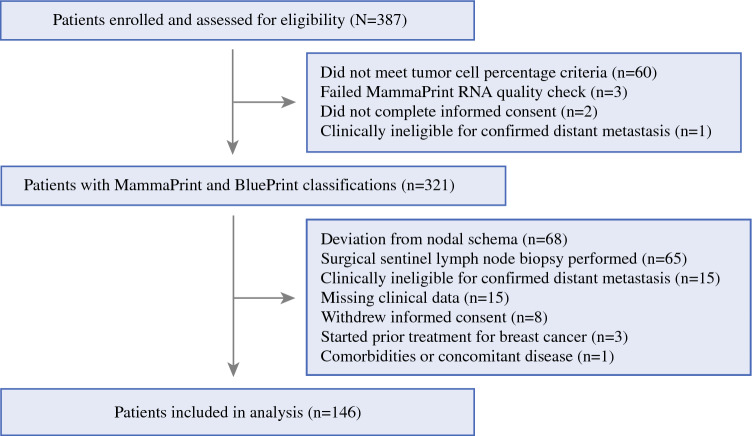
MINT consort diagram; patients with invasive breast cancer were prospectively enrolled from 2011 to 2016, this subanalysis includes 146 lymph node-positive patients

Patients (*N* = 387) aged ≥ 18 years with histologically confirmed invasive breast cancer were prospectively enrolled into MINT (NCT01501487) between 2011 and 2016 across 17 US institutions. Among the enrolled patients with T2–T4 invasive breast cancer, 321 had MammaPrint and BluePrint testing performed (Fig. 1). The current study is a post hoc subanalysis to evaluate nodal downstaging after neoadjuvant therapy. Only patients with node-positive tumors as confirmed on pretreatment biopsy were included in this subanalysis (*n* = 146; Supplementary Fig. 1). Preoperative nodal staging was assessed on core biopsy, and neoadjuvant therapy was given at the physician’s discretion, which aligned with established National Comprehensive Cancer Network (NCCN) guidelines. Upon completion of NCT, nodal involvement was assessed by ALND. This study was conducted in accordance with the ethical standards established in the Declaration of Helsinki. Institutional Review Boards at all participating sites approved of the protocol, and patient consent was obtained from all participants.

### Clinical and Molecular Subtyping

Preoperative core biopsies were used to assess hormone receptor (HR) and HER2 status by immunohistochemistry (IHC) or IHC/fluorescence in situ hybridization (FISH), respectively, per American Society of Clinical Oncology (ASCO)/College of American Pathologists (CAP) guidelines.^21,22^ IHC/FISH were performed under central pathology review at the University of South Florida. Clinical risk was determined using Adjuvant! Online (AOL) using MINDACT guidelines.^16^ The same core biopsies were used to isolate RNA for MammaPrint and BluePrint signatures, which were performed at Agendia (Irvine, CA, USA). Tumors were classified with MammaPrint as Low Risk (MammaPrint index > 0.000) or High Risk (MammaPrint index ≤ 0.000).^23^ BluePrint categorizes tumors by subtype (gLuminal, gHER2, or gBasal).^18^ Together, MammaPrint and BluePrint further stratified Luminal-Type tumors as gLuminal A (MammaPrint Low Risk) or gLuminal B (MammaPrint High Risk).^18^

### Objectives and Endpoints

The primary objective of the trial was to determine chemosensitivity prediction using MammaPrint and BluePrint. In MINT, therapeutic response was evaluated by pCR, defined as the absence of invasive carcinoma in the breast and axilla by microscopic examination of the resected specimen, regardless of the presence of carcinoma in situ (ypT0/Tis, ypN0). The objective of this post hoc subanalysis was to assess the ability of MammaPrint and BluePrint to predict the likelihood of nodal downstaging as well.

### Statistical Analysis

Patient clinical characteristics were summarized using descriptive statistics. Differences in age were evaluated by one-way analysis of variance (ANOVA), and other clinical characteristics were assessed by either chi-squared or Fisher’s exact test. The association of pCR or nodal downstaging with MammaPrint risk, combined MammaPrint and BluePrint subtype, or other clinical factors were assessed using a two-tailed proportional *z*-test. Statistical significance was defined by two-sided *p* < 0.05 for all tests. All statistical analyses were conducted using GraphPad Prism (version 9.0.2) and R (version 4.1.1).

## Results

### Patient Clinical Characteristics

The median age among the 146 node-positive patients was 53 years, with 56.3% post-menopausal patients (*n* = 142; Table 1). All patients were clinically high risk with intermediate (G2; 38.4%) to high grade (G3; 58.2%), cT2 (64.4%) and cN1 (87.0%) tumors. Of those with known receptor status (*n* = 145), 45.5% were HR+HER2− (*n* = 66), 20.0% HR+HER2+ (*n* = 29), 20.7% HR−HER2− (*n* = 30), and 13.8% HR−HER2+ (*n* = 20) by IHC/FISH. Overall, 15.8% (*n* = 23) of patients were MammaPrint Low Risk, and 84.2% (*n* = 123) were MammaPrint High Risk. Combined MammaPrint and BluePrint results classified the 146 patients as gLuminal A (*n* = 22; 15.1%), gLuminal B (*n* = 45; 30.8%), gHER2 (*n* = 38; 26.0%), and gBasal (*n* = 41; 28.1%).

**Table 1:** Patient clinical characteristics

	gLuminal A	gLuminal B	gHER2	gBasal	Total	*p*-Value
# of patients	22	45	38	41	146	
Age, median (SD)	55 (8.8)	57 (9.7)	44.5 (14.3)	53 (12.2)		0.166
Race/ethnicity						0.020
African American/Black	1 (4.5%)	6 (13.3%)	7 (18.4%)	14 (34.1.%)	28 (19.2%)	
Asian	0	0	3 (7.9%)	0	3 (2.1%)	
Caucasian/white	18 (81.8%)	36 (80.0%)	27 (71.1%)	24 (58.5%)	105 (71.9%)	
Hispanic	3 (13.6%)	3 (6.7%)	1 (2.6%)	2 (4.9%)	9 (6.2%)	
Native American	0	0	0	1 (2.4%)	1 (0.7%)	
Menopausal status						0.089*
Pre-	11 (50.0%)	13 (28.9%)	20 (52.6%)	18 (43.9%)	62 (42.5%)	
Post-	11 (50.0%)	32 (71.1%)	16 (42.1%)	21 (51.2%)	80 (54.8%)	
Unknown	0	0	2 (5.3%)	2 (4.9%)	4 (2.7%)	
Clinical subtype^a^						< 0.001*
HR+HER2−	19 (86.4%)	37 (82.3%)	1 (2.6%)	9 (21.9%)	66 (45.2%)	
HR+HER2+	2 (9.1%)	6 (13.3%)	20 (52.7%)	1 (2.4%)	29 (19.9%)	
HR−HER2−	1 (4.5%)	1 (2.2%)	1 (2.6%)	27 (65.9%)	30 (20.5%)	
HR−HER2+	0	0	16 (42.1%)	4 (9.8%)	20 (13.7%)	
Unknown	0	1 (2.2%)	0	0	1 (0.7%)	
Histologic grade						< 0.001*
Low, G1	1 (4.5%)	1 (2.2%)	0	2 (4.9%)	4 (2.7%)	
Intermediate, G2	17 (77.3%)	22 (48.9%)	13 (34.2%)	4 (9.8%)	56 (38.4%)	
High, G3	4 (18.2%)	22 (48.9%)	25 (65.8%)	34 (82.9%)	85 (58.2%)	
Unknown, GX	0	0	0	1 (2.4%)	1 (0.7%)	
Clinical T stage (cT)						0.764
cT2	16 (72.7%)	27 (60.0%)	25 (65.8%)	26 (63.4%)	94 (64.4%)	
cT3	6 (27.3%)	16 (35.6%)	10 (26.3%)	11 (26.8%)	43 (29.5%)	
cT4	0	2 (4.4%)	3 (7.9%)	4 (9.8%)	9 (6.2%)	
Clinical node status (cN)						0.185
cN1	17 (77.3%)	39 (86.7%)	36 (94.7%)	35 (85.4%)	127 (87.0%)	
cN2	5 (22.7%)	4 (8.9%)	2 (5.3%)	6 (14.6%)	17 (11.6%)	
cN3	0	2 (4.4%)	0	0	2 (1.4%)	
MammaPrint risk						< 0.001
Low risk	22 (100%)	0	1 (2.6%)	0	23 (15.8%)	
High risk	0	45 (100%)	37 (97.4%)	41 (100%)	123 (84.2%)	
Surgery type						0.241
Lumpectomy	10 (45.4%)	13 (28.9%)	8 (21.0%)	14 (34.2%)	45 (30.8%)	
Mastectomy	12 (54.6%)	32 (71.1%)	30 (79.0%)	27 (65.8%)	101 (69.2%)	

*SD* standard deviation, *HR* hormone receptor, *HER2* human epidermal growth factor receptor 2

*Patients with unknown clinical information were not included in chi-squared test

^a^HER2-equivocal designated as HER2-negative

### Nodal Downstaging with Neoadjuvant Chemotherapy and Association with Clinical Characteristics

**Fig. 2:**
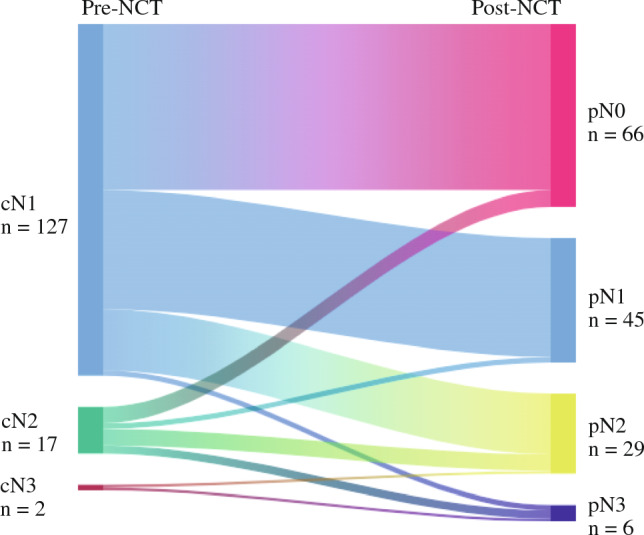
Nodal downstaging with neoadjuvant chemotherapy; Sankey diagram depicting the nodal status changes from pre-neoadjuvant chemotherapy (left) to post-neoadjuvant chemotherapy (right)

Most patients received taxane-based (*n* = 44; 30.1%) or taxane plus anthracycline or FEC-based chemotherapy (*n* = 100; 68.5%) with or without HER2-targeted therapy, per the physician’s discretion (Table 2). At the time of surgery, 45.2% (*n* = 66/146) of lymph node-positive patients had a nodal complete response (ypN0), of whom, 60.6% (*n* = 40/66) also achieved a complete response in the primary breast tumor, while 39.4% (*n* = 26/66) had residual disease in the primary tumor (Table 3). Notably, two patients with a breast-only complete response had residual axillary disease. The potential for nodal downstaging was inversely related to clinical lymph node status at diagnosis, where 47.2% (*n* = 60/127) of cN1, 35.3% (*n* = 6/17) of cN2, and 0% (*n* = 0/2) of cN3 patients were downstaged to ypN0, albeit not a statistically significant association (Fig. 2). There were three patients who had partial nodal downstaging (cN2 to pN1, or cN3 to pN2). In total, 27.4% (*n* = 40/146) of patients achieved a pCR. Of the patients with residual disease (*n* = 106), 26 (24.5%) had ypN0 downstaging despite not achieving resolution of the primary tumor (Table 3). Clinical factors associated with nodal downstaging were age (*p* = 0.037), menopausal status (*p* = 0.007), HR status (*p* = 0.025), and HER2 status (*p* < 0.001). In contrast, race/ethnicity (*p* = 0.790), preoperative clinical tumor size (*p* = 0.526), and clinical lymph node status (*p* = 0.434) were not associated with nodal downstaging (Table 4).

**Table 2:** Neoadjuvant treatment regimens

	gLuminal A	gLuminal B	gHER2	gBasal	Total
Patient number	22	45	38	41	146
Taxane based	4 (18.2%)	11 (24.4%)	26 (68.4%)	3 (7.3%)	44 (30.1%)
AC-T or FEC-T based	18 (81.8%)	33 (73.3%)	11 (28.9%)	38 (92.7%)	100 (68.5%)
Other^a^	0	1 (2.2%)^b^	1 (2.6%)^c^	0	2 (1.4%)

*AC* anthracycline + cyclophosphamide + taxane, *FEC* fluorouracil + epirubicin + cyclophosphamide + taxane

^a^Patient received endocrine therapy^b^ or AC (no taxane)^c^

**Table 3:** Nodal downstaging by MammaPrint and BluePrint subtype

Clinical nodal status by BluePrint	pCR	Residual disease				Total
	ypN0	ypN0	ypN1	ypN2	ypN3	
gLuminal A	0	4	10	8	0	22
cN1	0	4 (23.5%)	9 (52.9%)	4 (23.5%)	0	17 (77.3%)
cN2	0	0	1 (20.0%)	4 (80.0%)	0	5 (22.7%)
gLuminal B	5	7	15	14	4	45
cN1	4 (10.3%)	7 (17.9%)	15 (38.5%)	12 (30.8%)	1 (2.6%)	39 (86.7%)
cN2	1 (25.0%)	0	0	1 (25.0%)	2 (50.0%)	4 (8.9%)
cN3	0	0	0	1 (50.0%)	1 (50.0%)	2 (4.4%)
gHER2	22	6	8	2		38
cN1	21 (58.3%)	5 (13.9%)	8 (22.2%)	2 (5.6%)	0	36 (94.7%)
cN2	1 (50.0%)	1 (50.0%)	0	0	0	2 (5.3%)
gBasal	13	9	12	5	2	41
cN1	12 (34.3%)	7 (20.0%)	11 (31.4%)	4 (11.4%)	1 (2.9%)	35 (85.4%)
cN2	1 (16.7%)	2 (33.3%)	1 (16.7%)	1 (16.7%)	1 (16.7%)	6 (14.6%)

*pCR* pathological complete response, *cN* clinical nodal stage, *ypN* pathological nodal stage after neoadjuvant chemotherapy

**Table 4:** Association of nodal downstaging with genomic and clinical factors

	ypN0	RD	Chi-squared or Fisher’s exact test *p*-Value
Age, median*	48.5	57.0	0.037
Race/ethnicity			
African American/Black	12 (18.2%)	16 (20.0%)	0.790
Asian	2 (3.0%)	1 (1.3%)	
Caucasian/white	49 (74.2%)	56 (70.0%)	
Hispanic	3 (4.5%)	6 (7.5%)	
Native American	0	1 (1.3%)	
Menopausal status			
Pre-menopausal	35 (56.4%)	27 (43.6%)	0.007
Post-menopausal	27 (33.8%)	53 (66.2%)	
HR status			
HR+	37 (38.5%)	59 (61.5%)	0.025
HR−	29 (58.0%)	21 (42.0%)	
HER2 status			
HER2+	35 (71.4%)	14 (28.6%)	< 0.001
HER2−	31 (32.3%)	65 (67.7%)	
Tumor size (cT)			
cT2	46 (48.9%)	48 (51.1%)	0.526
cT3	17 (39.5%)	26 (60.5%)	
cT4	3 (33.3%)	6 (66.7%)	
Lymph node status (cN)			
cN1	60 (47.2%)	67 (52.8%)	0.434
cN2	6 (35.3%)	11 (64.7%)	
cN3	0	2 (100%)	
MammaPrint			
Low risk	4 (17.4%)	19 (82.6%)	0.007
High risk	62 (50.4%)	61 (49.6%)	
BluePrint			
gLuminal A	4 (18.2%)	18 (81.8%)	< 0.001
gLuminal B	12 (26.7%)	33 (73.3%)	
gHER2	28 (73.7%)	10 (26.3%)	
gBasal	22 (53.7%)	19 (46.3%)	

*Unpaired *t*-test

*ypN0* complete pathologic nodal downstaging after chemotherapy, *RD* residual disease, *HR* hormone receptor, *cT* clinical tumor stage, *cN* clinical node stage

### Identification of Nodal Downstaging by MammaPrint and BluePrint

**Fig. 3:**
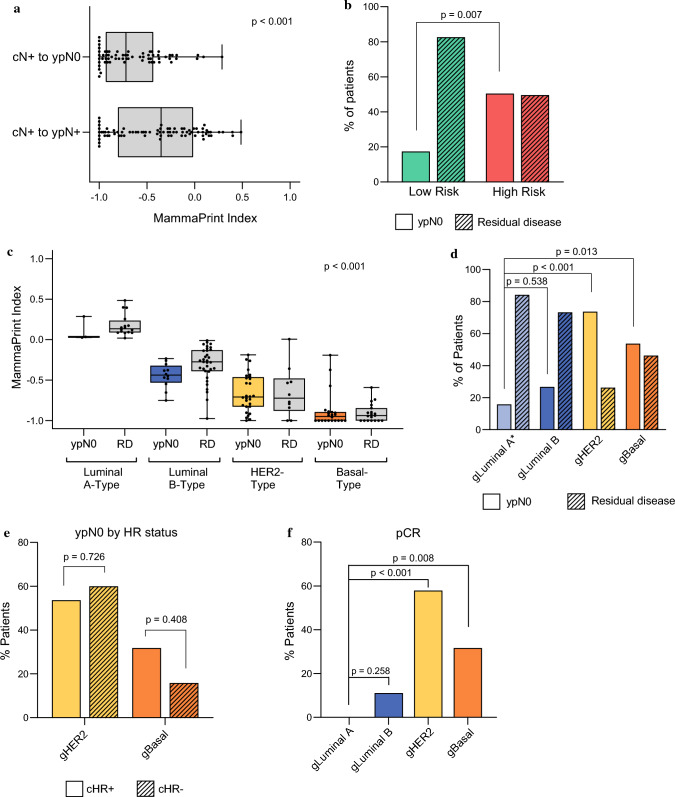
Association of nodal downstaging by MammaPrint and BluePrint; (**a**) MammaPrint Index was plotted for tumors from patients with nodal downstaging (cN+ to ypN0) and compared with residual disease tumors (cN+ to ypN+), (**b**) proportion of patients with Low Risk tumors having nodal downstaging (ypN0) and residual disease (RD) was compared with those with High Risk tumors, (**c**) patients were also categorized by BluePrint molecular subtype and MammaPrint Index was plotted for patients with ypN0 or RD for each molecular subtype, for gLuminal A tumors, MammaPrint indices are only shown for HR+HER2− tumors, (**d**) percentage of patients who achieved nodal downstaging for each BluePrint subtype, proportion of patients with gLuminal B, gHER2, and gBasal tumors who achieved ypN0 were compared with patients with gLuminal A tumors (the frequency of nodal downstaging for gLuminal A is shown for patients with HR+HER2− tumors), (**e**) within gHER2 and gBasal tumors, the percent of patients with nodal downstaging and residual disease are shown by hormone status, (**f**) percentage of patients who achieved pCR for each BluePrint subtype, proportion of patients with gLuminal B, gHER2, and gBasal tumors who achieved a pCR were compared with patients with gLuminal A tumors, significance between continuous variables (MammaPrint Index) was assessed using unpaired *t*-test or one-way ANOVA, two-tailed proportional *z*-test was used to compare categorical subgroups and between hormone status

To determine if nodal downstaging is associated with MammaPrint and BluePrint classifications, MammaPrint and combined MammaPrint and BluePrint results were compared with the number of patients with nodal downstaging versus those with residual nodal disease. MammaPrint was significantly associated with nodal downstaging when assessed as a categorical variable (*p* = 0.007; Table 4) as well as a continuous variable (*p* < 0.001; Fig. 3a). A larger proportion of patients with MammaPrint High Risk tumors achieved nodal downstaging compared with Low Risk tumors (*p* = 0.007; Fig. 3b). Of the patients with cN2–cN3 tumors (*n* = 19/146), 73.7% were MammaPrint High Risk (*n* = 14/19) and 26.3% were MammaPrint Low Risk (*n* = 5/19; Table 1). More patients with MammaPrint High Risk cN2–cN3 tumors had partial or complete nodal downstaging (57.1%; *n* = 8/14) compared with MammaPrint Low Risk cN2–cN3 tumors (20.0%; *n* = 1/5), albeit not significant (*p* = 0.365).

Combined MammaPrint and BluePrint results were also significantly associated with nodal downstaging when assessed as a categorical variable (*p* < 0.001; Table 4) and a continuous variable (*p* < 0.001; Fig. 3c). Among patients with HR+HER2−, genomically Luminal A-Type tumors, 15.8% (*n* = 3/19) had nodal downstaging. For patients with genomically High Risk tumors, 26.7% (*n* = 12/45) with gLuminal B, 73.7% (*n* = 28/38) of patients with gHER2, and 53.7% (*n* = 22/41) with gBasal tumors achieved complete nodal downstaging (ypN0; Fig. 3d). The proportion of patients with gHER2 and gBasal tumors that achieved nodal downstaging was significantly greater than patients with gLuminal A tumors (*p* < 0.001 and *p* = 0.013, respectively). There was a numerical difference (11%) in the proportion of patients who achieved nodal downstaging with gLuminal B tumors compared with gLuminal A tumors, albeit not significant (*p* = 0.538; Fig. 3d).

Interestingly, the proportion of patients with gHER2 and gBasal tumors who achieved ypN0 downstaging was not significantly different by HR status (*p* = 1.000 and *p* = 0.408, respectively; Fig. 3e). Similar to the rates of nodal downstaging, within the combined MammaPrint and BluePrint subtypes, 0% (*n* = 0/22) of patients with gLuminal A, 11.1% (*n* = 5/45) with gLuminal B, 57.9% (*n* = 22/38) of patients with gHER2 tumors, and 31.7% (*n* = 13/41) of those with gBasal tumors achieved a pCR. A greater proportion of patients with gLuminal B, gHER2, and gBasal tumors achieved a pCR compared with those with gLuminal A tumors (*p* = 0.258, *p* < 0.001, and *p* = 0.008, respectively; Fig. 3f).

## Discussion

Achieving a pCR has been associated with better outcomes and is considered by many to be a surrogate for long-term survival.^6,7,24^ Nodal downstaging, which can be achieved with NCT, has also been associated with better outcomes.^7^ pCR and nodal downstaging following NCT improves the rates of breast-conserving therapy and may de-escalate invasive surgical procedures. Accordingly, it is important to identify patients who may be candidates for receiving NCT and will likely achieve axillary downstaging to directly reduce surgery-associated morbidities. This is the first study using the genomic classifiers MammaPrint and BluePrint to identify patients who may be good candidates for less invasive surgical procedures such as TAD.

In this study, the rates of complete nodal downstaging (ypN0) were comparable to pCR rates for each combined MammaPrint and BluePrint subtype. No patients with gLuminal A tumors had a pCR in the breast and nodes, which is in line with results from recent NBRST and NBREaST-II studies.^19,25^ Although there are only a small number of patients with gLuminal A tumors included in this study, they exhibited the lowest rate of complete nodal downstaging with NCT in comparison with MammaPrint and BluePrint High Risk tumors. We observed variable responses in patients with gLuminal B tumors, with 11.1% achieving a pCR and 55.6% (*n* = 25/45) having a partial response in the primary tumor with NCT. Additionally, 26.7% of patients with gLuminal B tumors had nodal downstaging with NCT. Together, these data suggest that there is greater genomic diversity among patients with Luminal tumors, which can be distinguished with MammaPrint. Patients with gLuminal A and gLuminal B tumors both have underlying luminal biology, but gLuminal B tumors are considered High Risk with MammaPrint. Therefore, it is consistent with the characteristics of gLuminal B tumors to demonstrate lower response rates compared with other MammaPrint HighRisk tumor subtypes (gHER2 and gBasal) but to have higher response rates compared with gLuminal A tumors. These data also suggest that more effective preoperative therapies than endocrine therapy and standard chemotherapy are needed for patients with gLuminal B tumors. Thus, including these patients in trials evaluating new targeted therapies, such as CDK4/6 inhibitors or HER2 antibody-drug conjugates, should be considered. In contrast to patients with gLuminal A tumors, patients with MammaPrint High Risk or combined MammaPrint and BluePrint HER2-Type and Basal-Type tumors had significantly superior responses in the nodes and primary tumor. The majority of patients with gHER2 and gBasal tumors achieved complete nodal downstaging, of whom 78.6% (*n* = 22/28) and 59.1% (*n* = 13/22) also had a breast pCR, respectively. These results are consistent with previous studies in which more patients with clinically defined HER2+ and TN breast cancer achieve a pCR and nodal downstaging than with HR+HER2− or clinically-defined luminal tumors.^5,7,26,27^ Interestingly, the proportion of patients who achieved nodal downstaging within gHER2 and gBasal tumors were independent of HR status, which is in line with our recent report of patients with HR+/Basal tumors having distinct pCR rates and outcomes compared with patients with HR+/Luminal tumors.^20^ Importantly, there was a 15.8% discordance between clinical risk and MammaPrint risk assessment, where these clinically high-risk patients were identified as MammaPrint Low Risk. While MammaPrint is generally indicated for patients with cN1 disease, a higher proportion of patients with MammaPrint High Risk cN2–cN3 tumors achieved nodal downstaging (complete and partial) in comparison with MammaPrint Low Risk cN2–cN3 tumors, which is consistent with the cohort overall (including cN1). In patients with gLuminal A tumors, chemotherapy would still be indicated if surgical pathology was N2 or greater. Together, these data emphasize the expanded utility of using MammaPrint and BluePrint genomic testing, which can further characterize traditional clinical subtyping.

One limitation of this study is that we do not have long-term outcome data yet to correlate with BluePrint and MammaPrint results. If pCR is an accepted surrogate for long-term outcomes, we would expect that patients, particularly those with genomically HER2-Type and Basal-Type tumors who achieve pCR, will have a better prognosis compared with those with residual disease. This study is also limited by the small number of patients eligible for inclusion, and therefore a larger cohort would be recommended to confirm the results from this study.

## Conclusions

This is the first study to show that nodal downstaging can be predicted by MammaPrint and BluePrint beyond achieving a pCR or clinical factors such as HR status. Genomically High Risk patients, identified by MammaPrint with or without BluePrint molecular subtyping, had higher proportions of nodal downstaging than in the overall lymph node-positive cohort. Our data suggest that treating patients with clinically HR+HER2−, genomically Luminal A-Type tumors with chemotherapy for the purpose of nodal downstaging is likely to yield suboptimal results, and these patients should be counseled on this prior to treatment, whereas those with MammaPrint High Risk tumors respond better to NCT. Overall, results from this study suggest that MammaPrint and BluePrint may help identify good candidates for less invasive axillary procedures such as TAD, which may reduce surgery-associated complications and radiation and improve patient outcomes.

### Supplementary Information

Below is the link to the electronic supplementary material.

**FIG. S1** MINT nodal staging schema; patients enrolled into MINT followed the outlined staging schema, nodal involvement was established following neoadjuvant treatment by axillary lymph node dissection (ALND), for this subanalysis, lymph node-positive patients (n = 146) followed the teal-colored nodal staging and patients who received a surgical sentinel lymph node biopsy were excluded (DOCX 12 KB)
